# Can the Computed Tomographic Aspect of Porto-Systemic Circulation in Cirrhotic Patients Be Associated with the Presence of Variceal Hemorrhage?

**DOI:** 10.3390/medicina56060301

**Published:** 2020-06-19

**Authors:** Cosmin Caraiani, Bianca Petresc, Anamaria Pop, Magda Rotaru, Lidia Ciobanu, Horia Ștefănescu

**Affiliations:** 1 Department of Medical Imaging, “Iuliu Hațieganu” University of Medicine and Pharmacy Cluj-Napoca, 400012 Cluj-Napoca, Romania; ccaraiani@yahoo.com; 2 Department of Radiology, Regional Institute of Gastroenterology and Hepatology “Prof. Dr Octavian Fodor”, 400158 Cluj-Napoca, Romania; 3 Department of Radiology, Emergency Clinical County Hospital Cluj-Napoca, 400006 Cluj-Napoca, Romania; 4 Department of Radiology, “George Emil Palade” University of Medicine, Pharmacy, Science and Technology of Târgu Mureș, 540139 Târgu Mureș, Romania; 5 Department of Gastroenterology and Digestive Endoscopy, Medical Center of Gastroenterology, Hepatology and Digestive Endoscopy, 400132 Cluj-Napoca, Romania; anamaria.pop09@yahoo.com; 6 Department of Internal Medicine, “Iuliu Hațieganu” University of Medicine and Pharmacy Cluj-Napoca, 400012 Cluj-Napoca, Romania; rotaru.i.magda@gmail.com; 7 Department of Gastroenterology and Hepatology, Regional Institute of Gastroenterology and Hepatology “Prof. Dr Octavian Fodor”, 400158 Cluj-Napoca, Romania; ciobanulidia@yahoo.com (L.C.); drhstefanescu@gmail.com (H.Ș.); 8 Department of Gastroenterology and Hepatology, “Iuliu Hațieganu” University of Medicine and Pharmacy Cluj-Napoca, 400012 Cluj-Napoca, Romania

**Keywords:** cirrhosis, portal hypertension, variceal hemorrhage, CT scan, porto-systemic circulation, oesophageal varices, left gastric vein

## Abstract

*Background and objectives:* Variceal bleeding is a serious complication caused by portal hypertension, frequently encountered among cirrhotic patients. The purpose of this study was to determine whether the aspect of the collateral, porto-systemic circulation, as detected by CT are associated with the presence variceal hemorrhage (VH). *Materials and Methods:* 81 cirrhotic patients who underwent a contrast-enhanced CT examination were retrospectively included in the study. Patients were divided into two groups: Cirrhotic patients with variceal hemorrhage during the hospital admission concomitant, with the CT examination (*n* = 33) and group 2-cirrhotic patients, without any variceal hemorrhage in their medical history (*n* = 48). The diameter of the left gastric vein, the presence or absence and dimensions of oesophageal and gastric varices, paraumbilical veins and splenorenal shunts were the indicators assessed on CT. *Results:* The univariate analysis showed a significant association between the presence of upper GI bleeding and the diameters of paraoesophageal veins, paragastric veins and left gastric vein respectively, all of these CT parameters being higher in patients with variceal bleeding. In the multivariate logistic regression analysis, only the diameter of the left gastric vein was independently associated with the presence of variceal hemorrhage (OR = 1.6 (95% CI: 1.17–2.19), *p* = 0.003). We found an optimal cut-off value of 3 mm for the diameter of the left gastric vein useful to discriminate among patients with variceal hemorrhage from the ones without it, with a good diagnostic performance (AUC = 0.78, Se = 97%, Sp = 45.8%, PPV = 55.2%, NPV = 95.7%). *Conclusions:* Our observations point out that an objective CT quantification of porto-systemic circulation can be correlated with the presence of variceal hemorrhage and the diameter of the left gastric vein can be a reliable parameter associated with this condition.

## 1. Introduction

Portal hypertension develops when there is increased resistance to the portal blood flow [1]. The imaging hallmark of portal hypertension involves the development of portocaval collateral circulation, mainly of gastric and oesophageal varices. The prevalence of varices in patients with cirrhosis is 80–90% [2]. Variceal bleeding is one of the major causes of death among cirrhotic patients [3]. Mortality during the first episode of variceal bleeding is as high as 20–30% and it increases in patients with Child-Pugh C liver cirrhosis [4]. Patients with oesophageal or gastric varices develop hemorrhage at a rate of 10–30% per year [5]. Therefore, it is crucial to timely detect patients at risk for developing bleeding from gastro-oesophageal varices in order to perform proper therapies such as endoscopic ligation or transjugular intrahepatic portosystemic shunt [6]. Most patients with liver cirrhosis will undergo endoscopic screening for the presence of oesophageal varices [7]. The main predictors of variceal bleeding in clinical practice are: Large varices, red wale marks, Child-Pugh C liver cirrhosis [8].

Computed tomography (CT) is mainly used in cirrhotic patients for the detection and characterization of liver nodules, in order to rule out or confirm the presence of hepatocellular carcinoma (HCC). CT can also assess other complications of liver cirrhosis such as the presence of ascites, splenomegaly, portal thrombosis or sarcopenia.

Several studies have been published, proposing CT as a diagnostic tool for the evaluation of collateral circulation in cirrhotic patients [9,10,11,12,13,14,15,16]. The aim of our study was to determine if the aspect of the collateral, porto-systemic circulation, as detected by CT can be associated with the presence of variceal hemorrhage (VH). While most of the papers found in the literature focused on CT identification and grading of oesophageal and gastric varices, as compared to the endoscopic aspect and grading of oesophageal varices, our paper focused directly on identifying a CT pattern of the collateral, porto-systemic circulation, which can be associated with the presence of variceal hemorrhage.

## 2. Materials and Methods

The Ethics Committee of Regional Institute of Gastroenterology and Hepatology “Prof. Dr Octavian Fodor” Cluj-Napoca, Romania approved this retrospective study (5763/04.05.2020), waiving the informed consent from the patients. 

We performed a retrospective analysis of 110 patients with hepatic cirrhosis who underwent a CT examination in our institution between August 2017 and October 2018. We included in this study patients with an unequivocal diagnosis of liver cirrhosis who had an upper gastrointestinal (GI) endoscopy in a prior hospital admission or in the admission concomitant with the CT examination. The CT examination was done after the active bleeding was stopped and the patient was hemodynamically stable. The exclusion criteria are as follows: Patients without a prior endoscopy or without documented medical history (24 patients), patients who underwent non-enhanced CT scans and CT scans with inadequate quality for proper analysis (5 patients). Our final study population consisted of 81 eligible patients.

All CT examinations were performed in a single institution, using a 64-sliced CT machine (Siemens, 64 slices, Erlangen, Germany). In most cases a bi-phasic protocol was used after injection of contrast media, including an arterial and a portal phase. In some patients a tri-phasic protocol was used by adding a late phase. 

**Figure 1:**
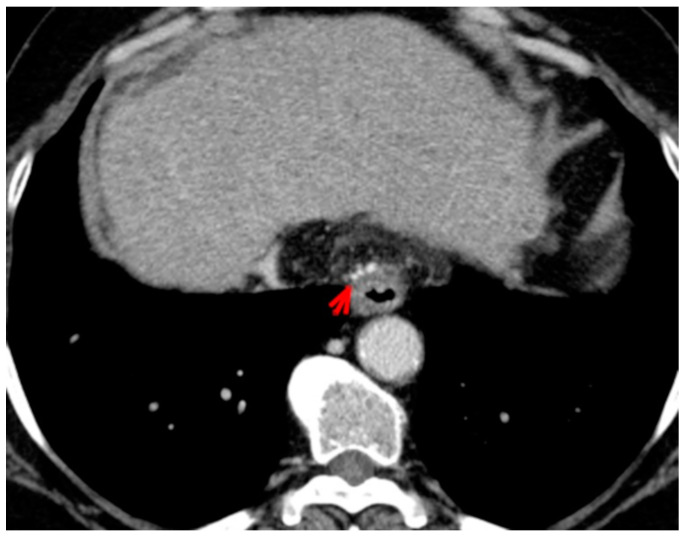
Axial contrast-enhanced CT shows enlarged paraoesophageal varices (red arrow).

**Figure 2:**
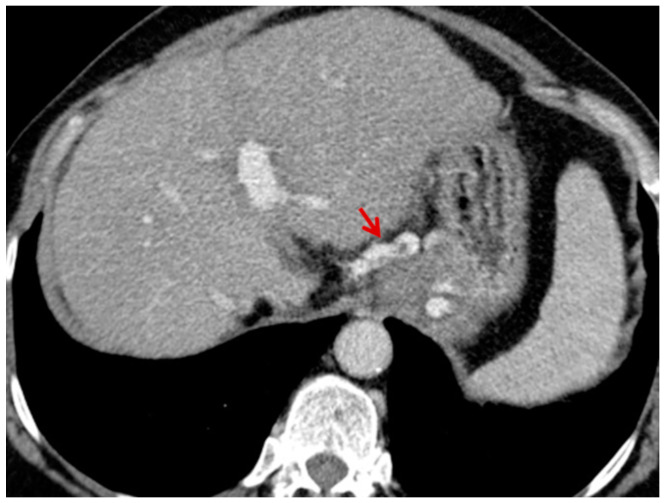
Axial contrast-enhanced CT shows enlarged paragastric varices (red arrow).

**Figure 3:**
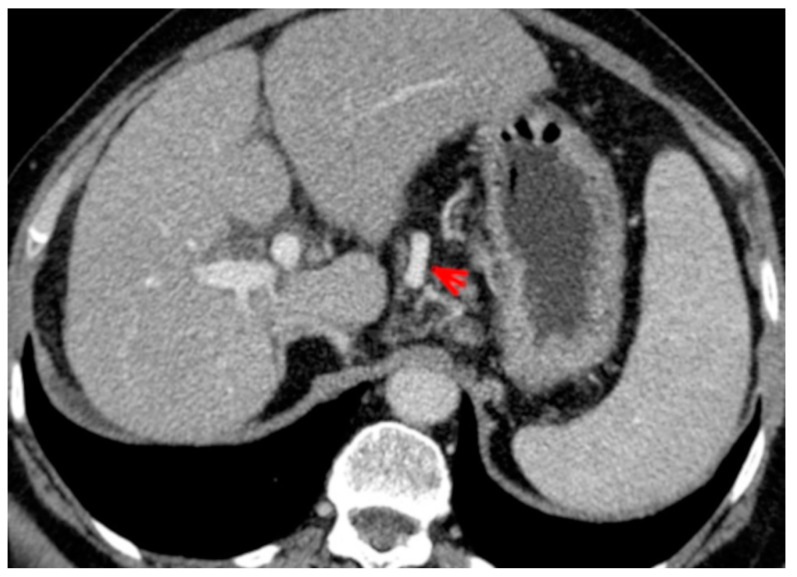
Axial contrast-enhanced CT shows enlarged left gastric vein (red arrow).

**Figure 4:**
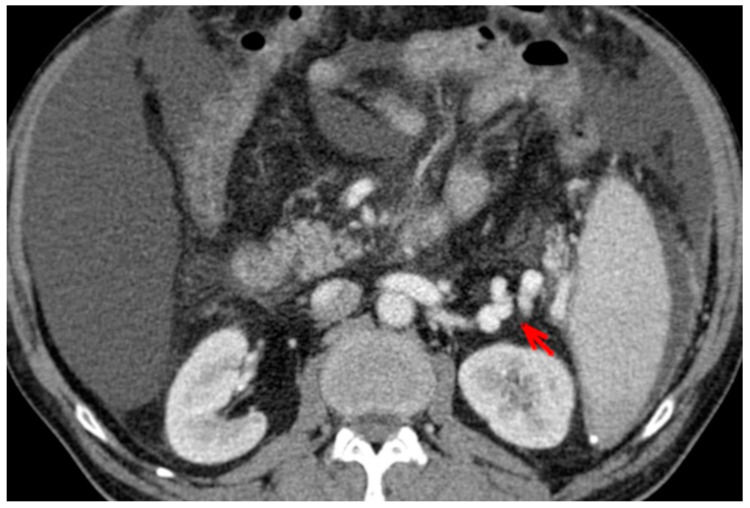
Axial contrast-enhanced CT shows spleno-renal shunt (red arrow).

**Figure 5:**
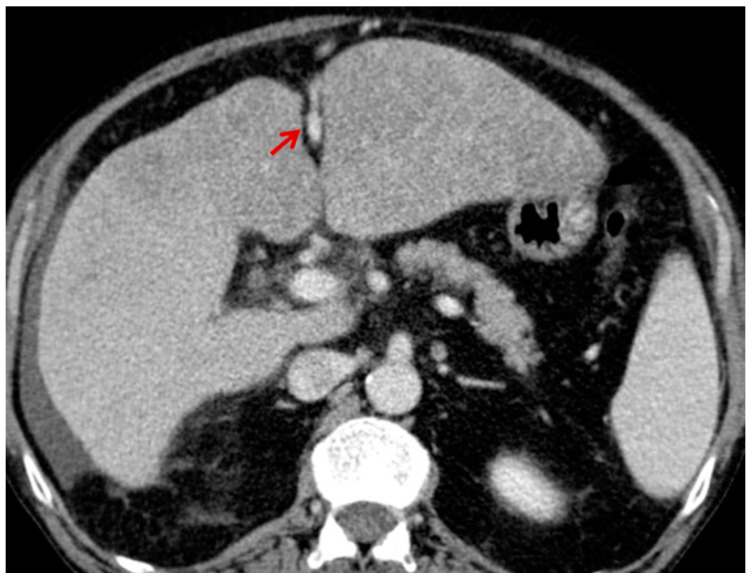
Axial contrast-enhanced CT shows repermeabilized round ligament (red arrow).

For this study, the images were retrospectively analyzed in consensus by two readers with experience in the field of abdominal imaging. The two readers assessed the following CT features: (1) The presence or absence of paraoesophageal varices and the size(mm) of the largest varix (Figure 1); (2) the presence or absence of paragastric varices and the size (mm) of the largest varix (Figure 2); (3) the largest diameter of the left gastric vein (Figure 3); (4) the presence or absence of splenorenal shunts and the size(mm) of the shunt (Figure 4); (5) the presence or absence of a repermeabilized round ligament and the size(mm) of it (Figure 5).

The total study population was divided into two groups: Group 1-patients with variceal hemorrhage from oesophageal varices (33 patients), group 2-patients without variceal hemorrhage (48 patients). Patients included in group 1 presented with VH at the emergency department, followed by a hospital admission during which the CT examination was performed. Group 2 consisted of patients with an upper GI endoscopy within 6 months prior to the CT examination, without any variceal bleeding in their medical history. 

Statistical analysis was performed with MedCalc for Windows, version 14.8 (MedCalc Software, Ostend, Belgium). Continuous variables with normal distribution were expressed as means ± standard deviation, those with non-normal distribution as median and 25th–75th percentile range. Normality of the data distribution was assessed by the Kolmogorov-Smirnov test. Categorical variables were expressed as absolute numbers and percentages. Normally distributed variables were compared using t–test. Non-normally distributed values were compared using Mann-Whitney test. Multivariate analysis (binary logistic regression model-enter method) was performed to identify independent predictors of variceal hemorrhage. Receiver operating characteristic (ROC) curve was performed to evaluate the accuracy of contrast-enhanced CT measurement of left gastric vein for the diagnosis of oesophageal hemorrhage. The diagnostic performance was expressed as the area under curve (AUC) with 95% confidence interval (CI), sensitivity with 95% CI, specificity with 95% CI, positive predictive value (PPV) with 95% CI, and negative predictive value (NPV) with 95% CI. The optimal cut-off value was chosen while the sum of sensitivity and specificity would be maximal. For all comparisons, a *p* value of < 0.05 was considered statistically significant. 

## 3. Results

The clinical and endoscopical characteristics of our study population are summarized in Table 1. The majority of the patients included in this study were males (66.7%). The most common etiology of the cirrhosis was alcoholic (43.2%). Approximately one third of the subjects included in this study had grade 2 oesophageal varices as diagnosed by endoscopy.

Our study population was divided into two groups: Group 1-patients presenting in the ER with VH (40.7%) and group 2-patients without VH in their medical history (59.3%). There was no significant difference between the two groups with respect to age and sex (*p* = 0.08). 

First, we performed a univariate analysis to determine if there is any association between several CT parameters and the presence of VH (Table 2). Our results show that the diameter of the paraoesophageal veins is significantly higher in patients with upper gastrointestinal (GI) bleeding (*p* = 0.02). Moreover, there was a significant association between the diameters of the paragastric veins and the presence of VH (*p* = 0.04). Furthermore, the diameter of the left gastric vein was demonstrated to significantly increase among the patients who had variceal bleeding (*p* < 001). However, our findings show no notable differences in the diameter of the falciform ligament or the diameter of spleno-renal shunts between the 2 groups of subjects (*p* = 0.97, and *p* = 0.72 respectively).

We performed a multivariate analysis in order to find which of these can be associated with a significant risk of presenting VH (Table 3). The logistic regression analysis shows that only the diameter of the left gastric vein can independently be associated with the risk of having upper GI bleeding (OR = 1.6 (95% CI: 1.17–2.19), *p* = 0.003).

**Figure 6:**
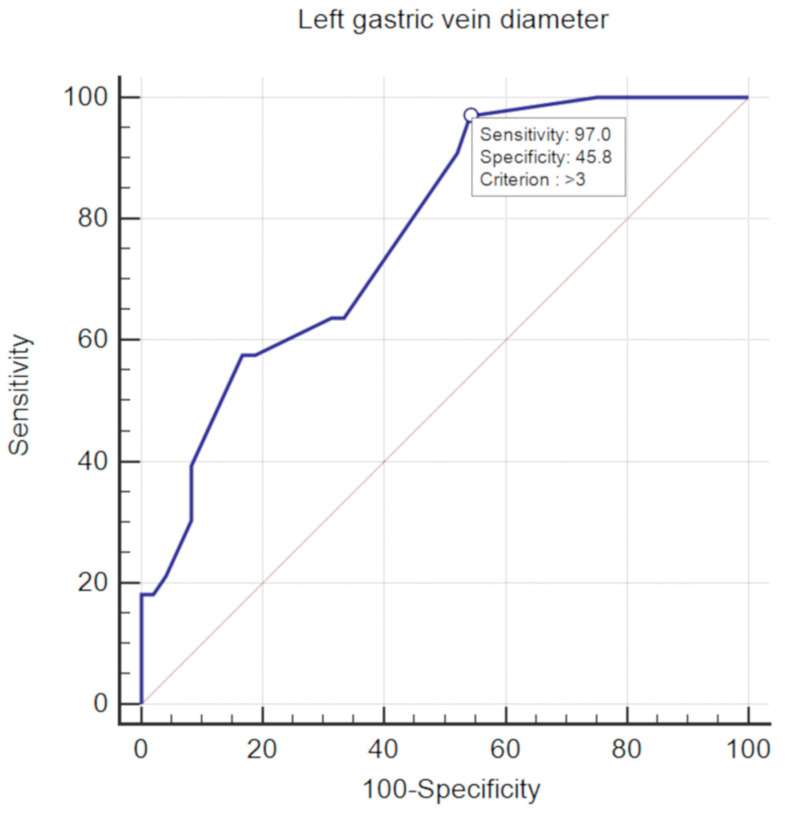
Receiver operating characteristic (ROC) curve of left gastric vein diameter measured on contrast-enhanced CT scan for the discrimination of patients with oesophageal hemorrhage.

Furthermore, we found an optimal cut-off value of 3 mm for the diameter of the left gastric vein (Figure 6) useful to discriminate among patients with variceal hemorrhage from the ones without it, with a good diagnostic performance (AUC = 0.78, 95% CI:0.674–0.864). The mean sensitivity and specificity for this cut-off value were 97% (CI: 84.2%–99.9%), and 45.8% (31.4%–60.8%), respectively, while the mean PPV and NPV were 55.2% (CI: 41.5%–68.3%), and 95.7% (CI: 78.1%–99.9%), respectively. 

Finally, our results indicate several strong positive associations between CT parameters and endoscopic grade of oesophageal varices (Table 4). We divided our population into two groups depending on the grade of oesophageal varices: group 1—with absent or grade 1 varices and group 2—with grade 2 or grade 3 varices. The diameters of paraoesophageal varices, paragastric varices and left gastric vein were significantly higher among patients with grade 2/grade 3 varices diagnosed by upper GI endoscopy. Still, the diameters of falciform ligament and spleno-renal shunts did not differ significantly between the 2 groups (*p* = 0.81, and *p* = 0.63 respectively).

## 4. Discussion

Upper gastrointestinal endoscopy is considered the gold standard for varices detection in liver cirrhosis. If varices are not found the patient will undergo endoscopic examination every two to three years for follow-up [17]. In patients with small, grade 1 varices, and compensated cirrhosis, endoscopy will be repeated every one to two years [7]. Despite the prophylactic treatment, there is a risk of progression of small varices to large varices and an increase of the hemorrhagic risk. Also new varices can be formed [8].

There is a group of patients with no worrisome features at the initial endoscopy, which will bleed from varices before the next endoscopy. The development of non-invasive techniques and markers to predict patients which are at risk for variceal bleeding may improve the outcome of these patients. Moreover, the identification of patients at risk of bleeding permits stratification in order to avoid potentially harmful treatments in the 60 to 75% of patients who will never have variceal bleeding [18].

Non-invasive methods to predict variceal grade and the chances of variceal bleeding have been studied [19]. A score including liver stiffness, spleen size and platelet count was proposed in order to identify patients with severe portal hypertension, at risk for developing variceal bleeding [20]. According to Baveno VI criteria, patients with liver stiffness < 20 kPa and platelet count over 150000/uL are at low risk (<5%) of developing VH and therefore do not require a screening endoscopy [18]. In addition to this our study suggests that patients with a left gastric vein with a diameter of 3 mm or less, as measured by contrast-enhanced CT, are at low risk of having VH and the diameter of the left gastric vein might be used as a non-invasive criteria of predicting variceal bleeding.

Most of the papers which analyze the correlation between the CT aspect of the porto-systemic circulation and the risk of variceal hemorrhage focus on the comparison between the diameter of the oesophageal varices, as assessed by CT, and the endoscopic aspect of the varices. 

The research of Shen et al. demonstrated a correlation between the CT aspect of oesophageal varices and the endoscopically assessed risk of variceal bleeding [19]. Findings in this paper are partially supported by our study, which shows a positive association between the diameter of oesophageal varices and the risk of developing variceal bleeding. In relation to the comparison between the CT measurement of oesophageal varices and their grading on upper GI endoscopy, our findings indicate a positive association, the diameters of oesophageal varices, measured on CT images, being significantly higher among patients with grade 2/grade 3 varices. 

These results are in concordance also with other papers which focused on the correlation between the aspect and grading of oesophageal varices on CT and upper digestive tract endoscopy. A research by Kim et al. proved a correlation between the size of oesophageal varices, measured by CT and the endoscopic grade of oesophageal varices [11]. A threshold of 3 mm diameter, measured by CT, can confidently separate large varices from small oesophageal varices, as detected by endoscopy [11]. Comparable results were obtained by another research group using a threshold of 2 mm to differentiate between small and large varices [12]. 

Dessouki et al. proposed the development of a computer assisted virtual esophagoscopy performed after oral ingestion of an effervescent powder in order to distend the oesophagus and make oesophageal varices easier to be detected and graded depending on their size and their hemorrhagic risk [13]. The correlations found between CT-oesophagography and endoscopy were very good with a diagnostic accuracy of 99% in defining oesophageal varices of all grades. However, in practice, CT scans in cirrhotic patients are, in most cases, made for other purposes and distension of the esophagus is not performed [14].

Some of the papers reported almost similar results of computed tomography and endoscopy in identifying and grading of oesophageal varices (grading of oesophageal varices includes appreciating the hemorrhagic risk). Howver, a recent metaanalysis which analyzed 11 previously published studies reports of an overall high misdiagnosis rate of 28.5%, in the CT diagnosis of oesophageal varices [15]. The studies concluded from the metaanalysis is that the CT results should be interpreted with caution due to the suboptimal specificity in the diagnostic and grading of oesophageal varices [15].

Few papers focus directly on the association between VH and the CT aspect and size of the collateral vessels. Somsouk et al. studied more parameters of portal hypertension, including the diameter of the left gastric vein. The studies found that the diameter of the largest oesophageal varix is the most reliable CT parameter in appreciating the risk of variceal bleeding [16]. This is in contradiction with our results which found that the diameter of the left gastric vein has a stronger association with VH than the diameter of the largest oesophageal varix. This may be due to the different designs of the two studies, Somsouk et al., including patients with an abdominal CT performed before the VH. In our series CT was performed after the VH and oesophageal varices may have collapsed after the hemorrhagic event, representing a possible explanation for the different results. However, the univariate analysis of Samsouk et al. revealed a significant association between the diameter of the left gastric vein diameter and the presence of VH, with a *p* value = 0.001 and OR = 3.47. 

Some papers proposed CT scores in order to appreciate the risk of developing variceal hemorrhage. Paul Calame et al. proposed a score combining the diameter of the paraumbilical vein and the splenic size as predictor of variceal bleeding. The results of this paper proved a significant negative association between the presence of a large paraumbilical vein (>5 mm) and the risk of developing variceal hemorrhage [21].

Another paper proposed a radiological score for identifying patients which are at risk for developing VH. The score includes diameters of the oesophageal varices, posterior gastric vein (diameter of this vessels positively correlate with the risk of hemorrhage from oesopageal varices) and diameters of the inferior mesenteric vein and short gastric vein (increase in diameter of these vessels will lead to a reduced probability of variceal hemorrhage) [22]. In our opinion this score is hard to reproduce in clinical practice due to difficulties in identification and correct measurement of the posterior gastric vein and of the short gastric vein (or veins). 

Our study is one of the few that compares CT indicators of portal hypertension, in order to classify their potential association with the presence of VH. The most reliable parameter was the diameter of the left gastric vein. Using a cut-off value of 3 mm, we achieved a very good sensitivity of 97% and a good predictive value of 95.6% for differentiating patients with VH. However, specificity and positive predictive value of this cut-off value are mediocre. 

Unlike other vascular structures such as oesophageal varices, short gastric or the posterior gastric vein, the left gastric vein is easily to be identified on contrast enhanced CT images. A study performed on healthy individuals proved that the left gastric vein is readily identifiable in 95.6% of patients (in the rest of the patients included in that study the quality of images was low) [23].

Our study is clinically relevant because it is, at our knowledge, the first paper which proves that diameter of the left gastric vein, as assessed by CT, can be associated with the presence of hemorrhage from oesophageal varices. Every increase in size by 1 mm of the left gastric vein is associated with 1.6 higher odds of presenting hemorrhage from oesophageal varices. Therefore, a dilated left gastric vein should put the patient at risk of developing VH and the patient should be monitored and treated accordingly.

Since one of the aims of our study was to determine whether contrast CT examination can be used as a non-invasive method for detecting patients at risk for variceal hemorrhage, excellent sensitivity and NPV values are more important, compared to specificity and PPV in a screening test. Considering the cut-off value of 3 mm diameter of the left gastric vein, we can use this as a screening measurement for further investigations such as upper GI endoscopy in cirrhotic patients. 

This study has several limitations. First, it is a retrospective study, which may lead to some bias in data collection and interpretation. Secondly, we did not exclude or separate patients, who were treated for their portal hypertension and VH risk with beta-blockers, or with endoscopic ligature of the varices. The treatment received by the patients may have an impact on the diameter of the collateral veins or on the diameter of the left gastric vein. However, patients treated with beta-blockers or with endoscopic ligatures were distributed into both groups of patients. Also, we did not exclude, neither separate, patients with hepatocellular carcinoma, portal vein thrombosis, sudden worsening or the liver function, acute superposing hepatitis or other factors, which could decompensate portal hypertension. 

Variceal hemorrhage can have an impact on the size of the porto-systemic collaterals. As we performed the CT studies in the first group of patients after the VH, we may have underestimated the size of the oesophageal varices and of the left gastric vein, as they may collapse after bleeding.

## 5. Conclusions

In conclusion, contrast enhanced CT can accurately appreciate collateral, porto-systemic pathways in patients with liver cirrhosis and portal hypertension. The most reliable CT parameter in differentiating patients at risk for developing upper digestive hemorrhage, due to variceal rupture from patients at low risk for VH is the diameter of the left gastric vein.

## Figures and Tables

**Table 1:** Clinical characteristics of the study population.

Variable	Value
Age ˜	59.4 ± 11.8
Male sex *	54 (66.7%)
Etiology or cirrhosis *	
Alcohol	35(43.2%)
HBV	9 (11.1%)
HCV	26 (32.1%)
Others	11 (13.6%)
Oesophageal varices *	
Absent	16(19.8%)
Grade 1	21 (25.9%)
Grade 2	29 (35.8%)
Grade 3	15 (18.5%)
Variceal hemorrhage *	
Present	33 (40.7%)
Absent	48 (59.3%)

˜ Results are presented as mean ± SD; * Results are presented as number (%).

**Table 2:** Comparison of the CT parameters between the two groups.

Variable	No VH	VH	*p* Value
Age	61.3 ± 11.1	56.7 ± 12.4	*p* = 0.08
Diameter of paraesophageal varices (mm)	2.5 (0.0–4.0)	4 (0.0–7.25)	*p* = 0.02 *
Diameter of paragastric varices (mm)	3 (0.0–5.0)	4 (0.0–8.25)	*p* = 0.04 *
Diameter of left gastric vein (mm)	2.5 (0.0–4.0)	6 (4.0–8.0)	*p* < 0.001 *
Diameter of falciform ligament (mm)	0 (0.0–3.87)	0 (0.0–4.5)	*p* = 0.97
Diameter of spleno-renal shunt (mm)	0 (0.0–2.25)	0 (0.0–3.5)	*p* = 0.72

* Statistically significant *p* < 0.05; Results are presented as mean ± SD, number (%), or median (25th–75th percentile); Abbreviations: VH: Variceal hemorrhage. The bold formatting to emphasize the statistically significant values of *p.*

**Table 3:** Logistic regression analysis for the association between CT measurements and presence of VH.

Variable	Odds Ratio (95% CI)	*p* Value
Diameter of left gastric vein (mm)	1.60 (1.17–2.19)	*p* = 0.0003 *
Diameter of paraesophageal varices (mm)	0.99 (0.82–1.21)	*p* = 0.99
Diameter of paragastric varices (mm)	0.99 (0.82–1.21)	*p* = 0.84

* Statistically significant *p* < 0.05. The bold formatting to emphasize the statistically significant values of *p.*

**Table 4:** Correlation between endoscopic grades of esophageal varices and CT measurements.

Variable	Absent/Grade 1	Grade 2/3	*p* Value
Diameter of paraesophageal varices (mm)	0 (0.0–4.0)	4 (0.0–7.37)	*p* = 0.01 *
Diameter of paragastric varices (mm)	2 (0.0–3.0)	4 (1.25–7.0)	*p* = 0.001 *
Diameter of left gastric vein (mm)	3 (2.25–6.0)	5 (4.0–7.75)	*p* < 0.001 *
Diameter of falciform ligament (mm)	0 (0.0–3.25)	0.0 (0.0–5.0)	*p* = 0.81
Diameter of spleno-renal shunt (mm)	0 (0.0–1.5)	0.0 (0.0–3.75)	*p* = 0.63

* Statistically significant *p* < 0.05; Results are presented as median (25th–75th percentile). The bold formatting to emphasize the statistically significant values of *p*.
